# Divergent Evolution of the Activity and Regulation of the Glutamate Decarboxylase Systems in *Listeria monocytogenes* EGD-e and 10403S: Roles in Virulence and Acid Tolerance

**DOI:** 10.1371/journal.pone.0112649

**Published:** 2014-11-11

**Authors:** Conor Feehily, Aiden Finnerty, Pat G. Casey, Colin Hill, Cormac G. M. Gahan, Conor P. O’Byrne, Kimon-Andreas G. Karatzas

**Affiliations:** 1 Bacterial Stress Response Group, Microbiology, School of Natural Sciences, College of Science, National University of Ireland, Galway, Galway, Ireland,; 2 Alimentary Pharmabiotic Centre and School of Microbiology, University College Cork, Cork, Ireland,; 3 Food Biosciences, University of Reading, Reading, United Kingdom; Institut de Pharmacologie et de Biologie Structurale, France

## Abstract

The glutamate decarboxylase (GAD) system has been shown to be important for the survival of *Listeria monocytogenes* in low pH environments. The bacterium can use this faculty to maintain pH homeostasis under acidic conditions. The accepted model for the GAD system proposes that the antiport of glutamate into the bacterial cell in exchange for γ-aminobutyric acid (GABA) is coupled to an intracellular decarboxylation reaction of glutamate into GABA that consumes protons and therefore facilitates pH homeostasis. Most strains of *L. monocytogenes* possess three decarboxylase genes (*gadD1, D2* & *D3*) and two antiporter genes (*gadT1* & *gadT2*). Here, we confirm that the *gadD3* encodes a glutamate decarboxylase dedicated to the intracellular GAD system (GAD_i_), which produces GABA from cytoplasmic glutamate in the absence of antiport activity. We also compare the functionality of the GAD system between two commonly studied reference strains, EGD-e and 10403S with differences in terms of acid resistance. Through functional genomics we show that EGD-e is unable to export GABA and relies exclusively in the GAD_i_ system, which is driven primarily by GadD3 in this strain. In contrast 10403S relies upon GadD2 to maintain both an intracellular and extracellular GAD system (GAD_i_/GAD_e_). Through experiments with a murinised variant of EGD-e (EGDm) in mice, we found that the GAD system plays a significant role in the overall virulence of this strain. Double mutants lacking either *gadD1D3* or *gadD2D3* of the GAD system displayed reduced acid tolerance and were significantly affected in their ability to cause infection following oral inoculation. Since EGDm exploits GAD_i_ but not GAD_e_ the results indicate that the GAD_i_ system makes a contribution to virulence within the mouse. Furthermore, we also provide evidence that there might be a separate line of evolution in the GAD system between two commonly used reference strains.

## Introduction

Survival in sometimes harsh environmental conditions is vital for any pathogen en route to infection of the host. The foodborne pathogen *Listeria monocytogenes* is well noted for an ability to withstand high salt environments [1]–[3], high pressure [4], [5], grow at low temperature [6] and within a broad pH range over which it can survive [3], [7], [8]. This makes it a major concern for the food industry where preservation methods often employ combinations of pH, salinity and temperature controls. In order for *L. monocytogenes* to survive low pH environments, the bacterium has evolved several mechanisms that allow it to maintain pH homeostasis. These include the arginine deiminase system [9], an F_0_F_1_ ATPase [10], the adaptive acid tolerance response (ATR) [11] and the glutamate decarboxylase (GAD) system [12]. The GAD system has been shown in *L. monocytogenes* to be important for survival in synthetic gastric fluid [12] but not in the presence of organic acids [8] commonly found in foods.

**Figure 1:**
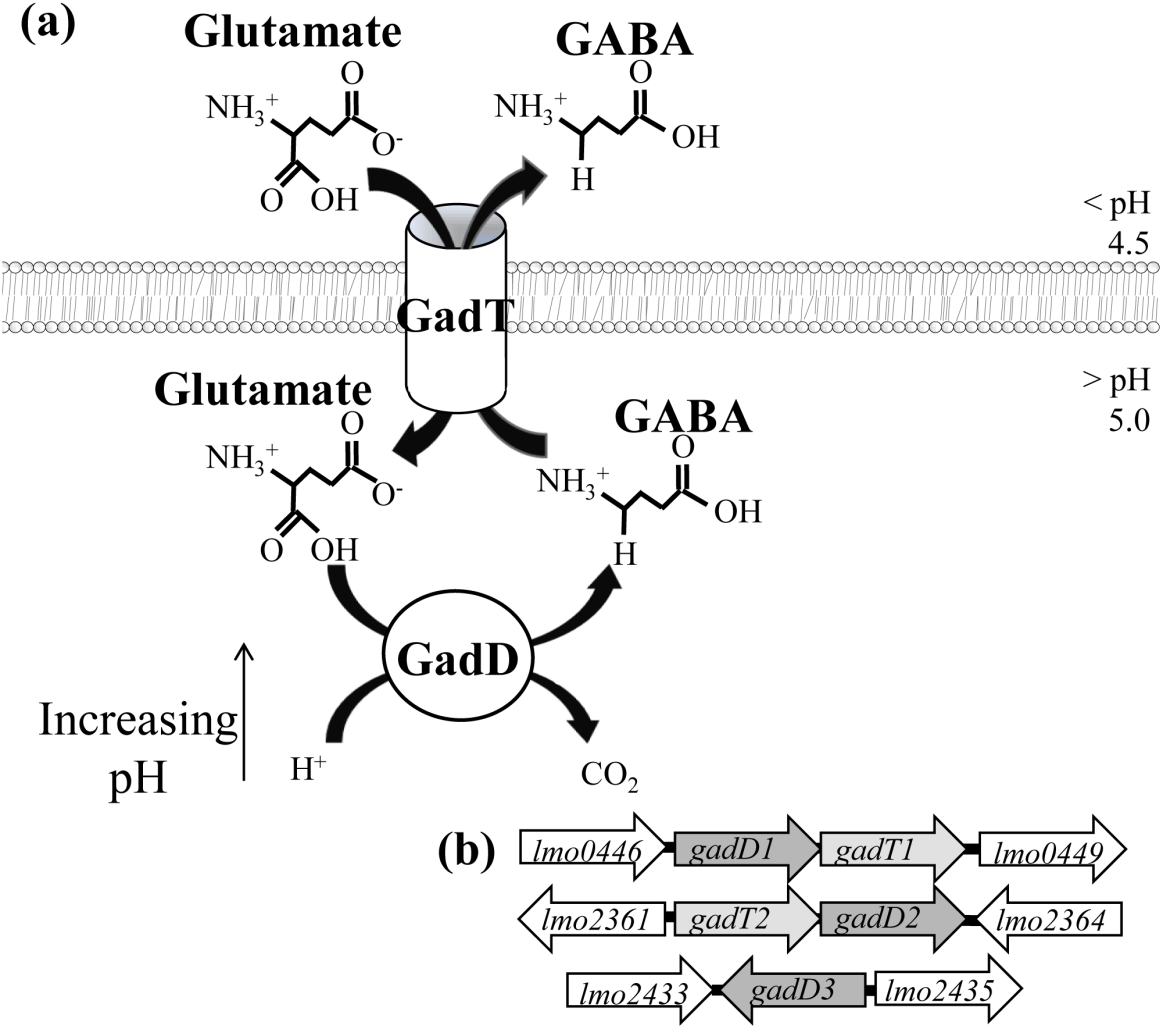
The standard model for the action of the GAD system. (**a**) A membrane bound antiporter carries glutamate into the cell in exchange for GABA. A cytosolic decarboxylase enzyme converts glutamate to GABA, with a consumption of H^+^. (**b**) The genomic structure of the genes encoding the GAD system in *L. monocytogenes* EGD-e.

The accepted model for the GAD system (Fig. 1) involves the combined action of a membrane bound antiporter (GadT) and a cytosolic glutamate decarboxylase (GadD). During exposure to low pH, the bacterium can exchange an extracellular molecule of glutamate for an intracellular molecule of γ-aminobutyric acid (GABA) via the GadT antiporter/s. This imported glutamate then undergoes a decarboxylation to form GABA via the GadD enzyme/s. At pH < 4.5, glutamate is imported in a neutral form (Glu^0^) [13], which allows the removal of intracellular H^+^ when glutamate is converted to GABA. This consumption of intracellular protons helps to maintain a tolerable intracellular pH. GABA generated via this reaction is expected to exit the cell via the antiporter in exchange for further glutamate, allowing a cycling process to continue (Fig. 1). In previous work we have shown that GAD activity can take place independently of the antiporter, a finding that prompted a revision of the previous model by introducing the concepts of an extracellular GAD system (GAD_e_) i.e. a GAD system relying on the Glu/GABA antiport and an intracellular GAD system (GAD_i_) i.e. a GAD system that relies on intracellular pools of glutamate or glutamate possibly imported into the cell via a glutamate transporter [14].

For *L. monocytogenes*, the glutamate/GABA antiporter can be encoded by one of two genes (*gadT1, T2*) while it possesses up to three decarboxylase encoding genes (*gadD1, D2 & D3*) [15]. Previously, *gadD3* has been considered as a putative glutamate decarboxylase encoding gene and some lines of evidence suggest that this glutamate decarboxylase may form an integral part of the GAD_i_ [14]. These genes are encoded in three transcriptional units *gadD1T1*, *gadT2D2* and *gadD3* [16] (Fig. 1). While all strains possess both *gadT2D2* and *gadD3*, strains from serotype 4 do not possess the *gadD1T1* operon [16]. Previously, genetic studies on the GAD system have shown that *gadD2* is critical for survival of *L. monocytogenes* at low pH, while *gadD1* is important at a milder pH [12], [16]. This work was carried out in the strain LO28 (serotype 1/2c), however recent work has shown that the GAD system can behave differently depending on both strain and media type. Analysis of strain EGD-e has shown that the antiporters are non functional, which results in a GAD system that produces intracellular GABA from the cytoplasmic pool of glutamate [14]. Furthermore, in a chemically defined media, the strain 10403S also relies solely on the activity of GAD_i_, despite having a functional antiport mechanism [17].

Despite what has been learned so far it is apparent that the accepted model of the GAD system in *L. monocytogenes* is not complete and certain knowledge gaps remain including elucidating the role of the GadD3 and of the other GAD genes in different environmental conditions as well as the role the GAD system plays *in vivo.* Therefore, here we compare the GAD system of two important reference strains of *L. monocytogenes*, EGDm (a murinised form of EGD-e) and 10403S and we describe an extensive genetic and physiological analysis of the GAD system in these strains, including the previously uncharacterised *gadD3* gene. Furthermore, while studies have shown that the GAD system is important for survival in porcine gastric juice, the role of this system during pathogenesis in a live animal model remains unknown.

## Methods and Materials

### Bacterial strains and growth

All strains used in this study are listed in Table 1. Strains were grown on BHI (Lab M) agar plates at 37°C for 24 to 48 h. Cultures grown overnight were set up by inoculating a single colony into 25 ml BHI broth in 250 ml Erlenmeyer flasks and incubating at 37°C with aeration.

**Table 1:** Bacterial strains and plasmids used in this study.

Strain or plasmid	Relevant properties	Reference/Source
*L. monocytogenes* strains		
EGDm	Serovar 1/2a, murinised *inlA* gene	C. Gahan
EGDm Δ*gadD1*	EGDm with in frame 1326 bp deletion of *gadD1* (*lmo0047*) gene	This study
EGDm Δ*gadD2*	EGDm with in frame 1365 bp deletion of *gadD2* (*lmo2363)* gene	This study
EGDm Δ*gadD3*	EGDm with in frame 1275 bp deletion of *gadD3* (*lmo2434)* gene	This study
EGDm Δ*gadD1D3*	EGDm Δ*gadD1* with in frame 1275 bp deletion of *gadD3* (*lmo2434)* gene	This study
EGDm Δ*gadD2D3*	EGDm Δ*gadD3* with in frame 1365 bp deletion of *gadD2* (*lmo2363)* gene	This study
10403S	Serovar 1/2a, Wild-type	K. Boor
10403S Δ*gadD1*	10403S with in frame 1326 bp deletion of *gadD1* (*lmrg_00139*) gene	This study
10403S Δ*gadD2*	10403S with in frame 1365 bp deletion of *gadD2* (*lmrg_01479)* gene	This study
10403S Δ*gadD3*	10403S with in frame 1275 bp deletion of *gadD3* (*lmrg_01814)* gene	This study
Plasmid		
pKSV7	pKSV7 shuttle vector used to carrying the individual deletion cassettes used in mutant generation	[34]

### Generation of knockout mutants

Deletion mutants were generated using the splicing by overlap extension (SOEing) PCR method and allelic replacement, as previously described [18]. All primers used in the process are listed in Table 2. Deletion cassettes were cloned into the shuttle vector pKSV7. Double mutants were generated by introducing the deletion cassette into the confirmed single EGDm Δ*gadD3* mutant. PCR amplification of DNA for use in cloning and downstream work was carried out by using the high-fidelity Velocity DNA polymerase (Bioline), while screening was carried out by using Biotaq DNA polymerase (Bioline).

**Table 2:** Primers used in this study.

Primer name	Sequence (5' to 3')
*gadD1* A	CGGAATTCCAAGCAAATTACCAGGTG
*gadD1* B	GAGTAAAGCCAGAGCCAAACACCGGTACA
*gadD1* C	CGGTGTTTGGCTCTGGCTTTACTCATTAAG
*gadD1* D	CGGAATTCATAACGTAAGATAGTGCGCC
*gadD1* For	ACAAATACGCCACGCATC
*gadD1* Rev	GGCAAGAACCATAAGAATCCAC
*gadD2* A	CGGAATTCGTAGTCATTTATTTAGTCGGC
*gadD2* B	AAGCCGTGACTATATAACATGATTTTTTCCTC
*gadD2* C	TATATAGTCACGGCTTCACACATTAATAAAAAGGC
*gadD2* D	CGGAATTCTCGCATATTAATTATTTGACG
*gadD2* For	TCATTCCTAACTGCCATTTCC
*gadD2* Rev	TGGAATGAGAATAGTGGACGG
*gadD3* A	CGGAATTCCTTTATAGTGAAGACGAC
*gadD3* B	TTGTCATGATACATACAAGCTTCCGAAG
*gadD3* C	GCTTGTATGTATCATGACAAAGAACGCAAC
*gadD3* D	TAGAATTCATTTCAGTACGCGAGCCATCAC
*gadD3* For	GAACCTCCTTATAAGTACCATC
*gadD3* Rev	GGTGGTTACGGTGCATTC

### Acid survival assays

Cultures of bacteria were grown to mid-exponential (OD_600_ = 0.35) or stationary phase (16 h) at 37°C in BHI medium. The pH of these cultures was lowered with 3 M HCl to pH 3.5 for mid-exponential or pH 2.5 for stationary phase cultures. Samples were taken every 20 min for 1 h and serially diluted in phosphate-buffered saline (PBS). Dilutions were plated in triplicate onto BHI agar and incubated overnight at 37°C. Colonies were counted to determine the number of surviving cells.

### GABase assays

Intracellular GABA (GABA_i_) and extracellular GABA (GABA_e_), were measured as previously described [17], [19]. Cultures were grown to mid-exponential phase (OD_600_ = 0.35) or stationary phase (16 h) at 37°C with aeration in BHI medium. For exponential phase GABA measurements the cultures were reduced to pH 4.0, while for stationary phase GABA measurements, the pH of the cultures was lowered to 4.0 (EGDm) or 3.5 (10403S) with 3 M HCl. Different pH reductions for each strain were necessary to ensure optimal GABA production for each strain. Extractions were made after 1 h of acid treatment. Non HCl-treated cultures were used as negative controls. GABase from *Pseudomonas fluorescens* (Sigma Aldrich, Steinheim, Germany) was used in the enzymatic assay and increases in OD_340 nm_ were measured using a Tecan Sunrise absorbance plate reader.

### Real-time PCR determination of *gadD* gene transcription

Real-time PCR was used to determine the relative expression of the genes encoding the GAD system, using the 16S rRNA gene as a reference gene. Cells were grown overnight (16 h) in triplicate (biological replicates) in 20 ml BHI at 37°C. Prior to RNA isolation, 1 ml of culture was mixed with 2 ml RNA*later* ^(R)^ (Sigma) and incubated at room-temperature for 5 min. Subsequently cells were harvested by centrifugation at 8,000×g for 5 min washed once in 1 ml 1 M Tris buffer (pH 7.4) and disrupted by microwaving for 15 s at 700 W. Pellets were resuspended in 350 µl Buffer RLT (Qiagen) and cell debris removed by centrifuging at 12,000×g for 2 min. Total RNA was obtained from each of the biological replicates using the RNeasy kit (Qiagen, Hilden, Germany), quantified using a NanoDrop (Thermo Scientific, Wilmington, DE), treated with DNase (Turbo DNA-free; Ambion, Austin, TX), and then used to synthesize cDNA. To ensure the absence of DNA contamination in the samples of treated RNA, a PCR reaction was carried out on each RNA sample using 16S primers. To obtain cDNA 15 µl of total RNA (2 µg ml^−1^) was mixed with 1 µl of random primers (3 µg µl^−1^; Invitrogen, Carlsbad, CA), 1 µl of a 10 mM stock of deoxyribonucleotide (dNTP) mix (Invitrogen, Carlsbad, CA). The mixture was heated to 65°C for 5 min, transferred onto ice, and centrifuged briefly before 4 µl of First Strand buffer and 2 µl or 100 mM dithiothreitol (DTT) was added. Following an incubation of 2 min at 25°C, 1 µl of Superscript II (200 U µl^−1^) was added. Subsequently, the mixture was incubated at 25°C for 10 min, at 42°C for 50 min, and finally at 70°C for 15 min before the cDNA was stored at −20°C. Relative quantification of the expression of the genes was carried out by fluorometric real-time PCR using the SYBR green master mix and the Quantitect SYBR green PCR kit (Qiagen, Hilden, Germany). Following the instructions of the kit, 2.5 µl of cDNA was mixed with 5 µl QuantiTect SYBR green PCR master mix (containing fast-start Taq DNA polymerase, SYBR green dye, buffer, and MgCl_2_), and the appropriate set of primers (Table 1) was added to a final concentration of 0.2 µM each. Each set of primers was designed with specificity toward the genome of *L. monocytogenes* strain 10403S (accession no. AARZ02000000), producing amplicons in the range of 207 to 268 bp (Table 1). Finally, molecular-biology-grade water was added to the mixture, up to a final volume of 10 µl, and placed in a well of the 96-well microtiter plate (LightCycler 480 multiwell plate 384; Roche Diagnostic GmbH, Manheim, Germany). Once the other PCRs were set up in the other wells, the plate was placed in the LightCycler 480 instrument (Roche Diagnostic GmbH, Manheim, Germany), and the PCR was programmed starting with an initial denaturation at 95°C for 10 min, followed by amplification for 40 cycles at 95°C for 1 s, 5 s at various annealing temperatures, depending on the melting temperature of the set of primers (Table 1), and 72°C for 9 to 11 s.

The specificity of amplification for each product was determined by a melting curve analysis at 95°C for 1 s and 65°C for 15 s, followed by a progressive increase of the temperature to 95°C with a ramp rate of 0.11°C s^−1^, with continued measurement of fluorescence, and finally cooling of the plate at 40°C for 30 s. Alongside each real-time PCR assay, a control reaction without added cDNA was run as a negative control. Relative expression was calculated as a ratio between expression of target genes (*gadD1, gadT1, gadD2, gadT2,* and *gadD3*) and the expression of the 16S rRNA gene, which served as reference gene in each cDNA sample. Calculations were carried out following the “advanced relative quantification” settings of the LightCycler 480 software program with PCR efficiency correction.

### Animal and infection studies

Eleven week old Balb/c mice were used for infection studies. Animals were housed within the biological services unit, University College Cork and fed 2018S Teklad Global 18% Protein Rodent Diet (Harlan). The EGDm strain used in this study was murinised as previously described [20]. All GAD system mutants were introduced into this strain prior to infection. Overnight cultures of bacteria were grown in BHI medium at 37°C for 16–18 h. Cultures were washed and resuspended in PBS. Mice were inoculated with bacteria to a concentration of 10^10^ in 200 µl via direct gastric gavage at 5 mice per strain. Three days post infection, mice were sacrificed and the liver, spleen, mesenteric lymph nodes (MLN) and intestinal faeces were harvested. Liver, spleen and MLN were homogenised in PBS. The weight of the intestinal content was determined and then homogenised in PBS. All samples were serially diluted and plated in duplicate onto either BHI agar (Liver & Spleen) or Listeria Selective agar (LSA; Oxoid) (MLN & intestinal content).

### Macrophage survival assay

Permanent stocks of a human derived THP-1 macrophage cell line [21] were maintained at a concentration of 1×10^6^ in 1 ml Roswell Park Memorial Institute (RPMI) 1640 medium (Sigma Aldrich) supplemented with 10% (v/v) heat-inactivated foetal bovine serum (FBS; Lonza) and 10% (v/v) DMSO and stored at −80°C. These were recovered by resuspension in 3 ml RPMI supplemented with 10% (v/v) FBS and 100 U ml^−1^ penicillin/streptomycin (PenStrep; Sigma Aldrich) and incubated at 37°C in 5% CO_2_ for 2 days. The culture was then centrifuged at 400×*g* for 5 min and the pellet washed in 5 ml phosphate buffered saline and centrifuged again. The pellet was then resuspended in 5 ml RPMI with 10% FBS and PenStrep and incubated as above for 5–7 days. For survival assays, the macrophages were centrifuged at 400×*g* for 5 min and washed twice in 5 ml PBS and resuspended in RPMI and 10% FBS supplemented with 0.16 µM phorbol myristate acetate to a concentration of 1×10^5^ cells ml^−1^. One millilitre was then seeded per well of a 24 well tissue culture plate and the plate was incubated for a further 24 h at 37°C with 5% CO_2_ to allow the cells to adhere to the surface of the well. After incubation, the media was removed from each well and 1 ml of PBS was added to wash the cells. The plate was shaken gently and the PBS removed. In parallel, *L. monocytogenes* cultures were grown to stationary phase in BHI where 1 ml of culture was then centrifuged at 10,000×*g* for 5 min, washed once in 1 ml PBS and resuspended in 1 ml PBS to give an estimated concentration of 1×10^9^ cfu ml^−1^. Bacterial cells were then diluted to 1×10^6^ in RPMI and 10% FBS. One millilitre of this bacterial suspension was added to each washed well of THP-1 cells to give a multiplicity of infection (MOI) of 10. Plates were incubated at 37°C with 5% CO_2_ for 1 h to allow for invasion. After the 1 h co-incubation period, the media was removed from the wells and the cells were washed with 1 ml PBS and then 1 ml of RPMI and 10% FBS supplemented with 30 µg ml^−1^ Gentamicin was added to kill remaining extracellular bacteria. Plates were returned to incubate for 1 h at 37°C with 5% CO_2_. Bacterial counts were performed by removing the media from each well, washing once in 1 ml PBS and resuspending the cells with 1 ml ice cold sterile dH_2_O for 1 min. Serial dilutions were performed and the suspension was spread plated in duplicate onto BHI agar plates. All agar plates were incubated for 24 h at 37°C before counting.

### Statistical analysis of results

Experiments were carried out with three biological replicates and at least two technical replicates for each sample. Significant differences between samples tested were determined by using either a paired Student *t* test or one-way ANOVA. Results were considered significant when they possessed a P value of <0.05. Error bars indicating standard deviations from the means are displayed on graphs.

### Ethical statement

All animal procedures were approved by the University Animal Experimental Ethics Committee (AEEC) in University College Cork (approval ID 2011/017) and were carried out in a specialized facility. Mice were euthanized by cervical dislocation. Work was carried out under license from the Irish Department of Health.

## Results

### Deletion of *gadD1* or *gadD2* increases acid survival in EGDm

**Figure 2:**
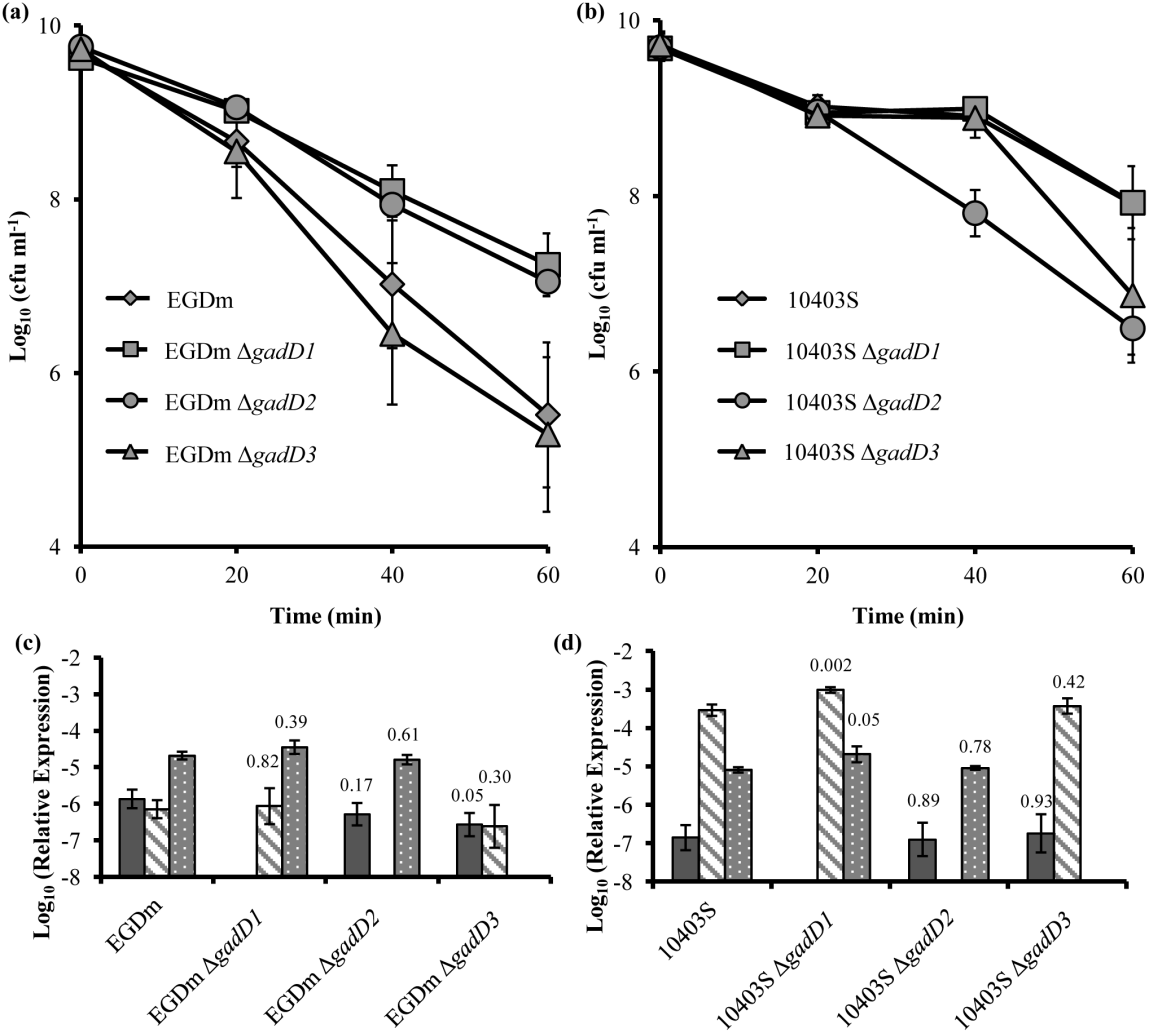
Acid survival of *L. monocytogenes gad* mutants. Stationary phase EGDm (**a**) and 10403S (**b**) Δ*gad* mutants were challenged at pH 2.5. Cell counts were taken every 20 min. Values are the means of data from three individual cultures, with the cell counts for each culture being the means of counts from three platings. Relative transcript levels of EGDm **(c)** or 10403S (**d**) *gadD1* (dark grey fill), *gadD2* (hatched) and *gadD3* (grey) genes to 16S gene prior to acid exposure in each mutant strain. Error bars represent the standard error from the mean value of three individual biological repeats. The numbers over the bar charts (**c & d**) indicate the *p*-value for the difference between each gene expression compared to wild-type levels as determined by student’s *t*-test.

To investigate the role of the GAD system in acid tolerance and virulence each of the three decarboxylase genes (*gadD1*, *gadD2*, *gadD3*) were deleted individually from a murinised strain of EGD-e (designated EGDm), generating three deletion mutants. The murinisation process as explained and carried out previously [22], involved the targeted mutagenesis of two key amino acids (Ser192Asn and Tyr369Ser) in the InlA protein of EGD-e. Strains lacking a single *gadD* gene were grown to stationary phase (at which point the GAD system is known to play a crucial role in the survival of acidic conditions [12], [23]) and tested for survival at low pH. After 20 min the numbers of surviving wild-type EGDm cells began to rapidly reduce as did those of the strain with a deletion in *gadD3.* Sixty minutes post treatment both the parental EGDm and EGDm Δ*gadD3* had reduced by over 4.5 log-cycles (Fig. 2**a**). In contrast the numbers of both EGDm Δ*gadD1* and EGDm Δ*gadD2* strains reduced by only ∼2 log-cycles after 60 min. When the survival of mid-exponential phase cells was tested the *gadD1* and *gadD2* deletions did not confer any survival advantage compared to the parental control (Fig. S1**a**). Measurements of the stationary phase transcript levels of each *gadD* gene prior to acid exposure showed that *gadD3* was expressed by almost 2 orders of magnitude greater than either *gadD1* or *gadD2* (Fig. 2**c**). Deletion of any of the *gadD* genes did not appear to significantly affect the base levels of any of the remaining two *gadD* genes although *gadD1* in the Δ*gadD3* background was reduced but this difference displayed a *p*-value of 0.05. Thus the increased stationary phase acid resistance observed in the EGDm Δ*gadD1* and EGDm Δ*gadD2* strains cannot be explained by an increase in the transcription of the remaining *gadD* genes.

### Deletion of *gadD2* and *gadD3* reduces acid tolerance in 10403S

To determine if the relative contribution of the *gadD* genes to acid tolerance was conserved in another well-studied reference strain the same gene deletions were introduced into *L. monocytogenes* 10403S, which belongs to the same lineage (II) as EGD-e, but carries over 30,000 SNPs [24]. Once more the effect of acid treatment was tested on stationary phase cultures for 1 h in BHI. After 60 min wild-type 10403S showed a reduction in cell numbers of over 1.5 log-cycles (Fig. 2**b**), substantially less killing than was observed in EGDm over this period (Fig. 2**a**). Deletion of *gadD1* did not affect survival at this pH compared to the wild-type. For 10403S a steady decrease in cell numbers was observed over 60 min with a final reduction of about 3 log-cycles, 100-fold greater than the parent strain. The increased sensitivity of the Δ*gadD2* mutant was also observed in exponentially growing cells (Fig. S1**b**). 10403S Δ*gadD3* did not show a significant reduction in cell numbers compared to 10403S until after 40 min of acid treatment and reached a final reduction of about 3 log-cycles after 60 min (Fig. 2**b**). A profile of the *gadD* gene transcript levels prior to acid exposure in 10403S showed that *gadD2* is the dominant transcript while *gadD1* is transcribed at the lowest levels (Fig. 2**d**). As in EGDm, deletion of one *gadD* gene did not significantly affect the expression of the remaining genes apart from *gadD2* in the Δ*gadD1* background. Together these results suggest that the two strains have evolved different transcriptional controls over the *gadD* genes and further indicate that *gadD* gene transcript levels *per se* don’t determine the intrinsic level of acid tolerance.

### EGDm relies on *gadD3* for GABA production while 10403S requires *gadD2*

**Figure 3:**
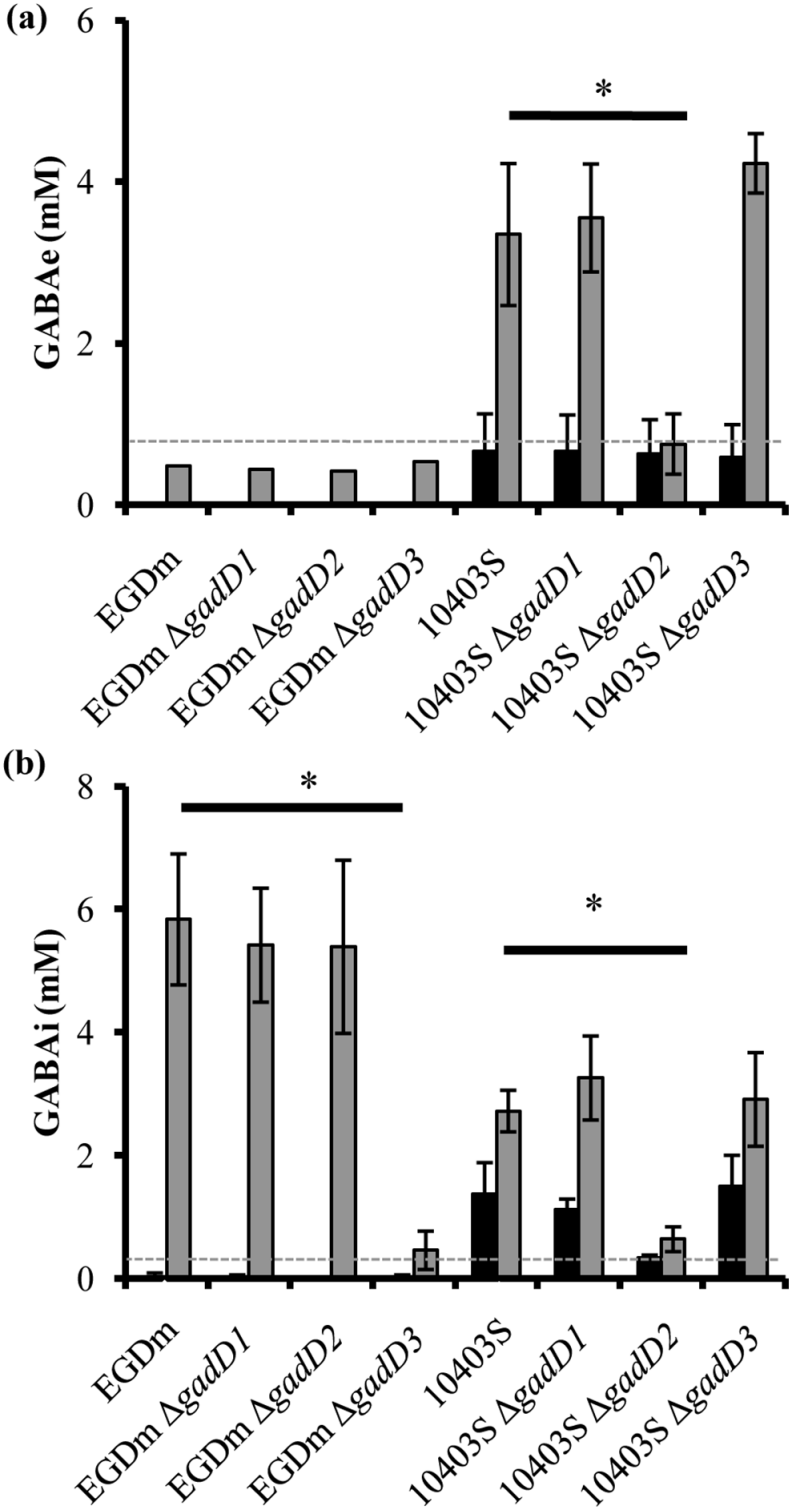
GABA production from *L. monocytogenes gad* mutants. (**a**) Production of GABA_e_ by EGDm and 10403S gad mutants with (grey) or without (black) 1 h exposure to acid at pH 4.0 (EGDm) or pH 3.5 (10403S). (**B**) Production of GABA_i_ by EGDm and 10403S *gadD* mutants with (grey) or without (black) 1 h exposure to acid at pH 4.0 (EGDm) or pH 3.5 (10403S). Dashed horizontal lines indicate the detection limits for GABA in each experiment. Error bars represent the standard deviation from the mean of three individual biological repeats for each sample. An asterix represents signifcant difference of less than 0.05 between a given mutant and respective wild-type as determined by a student’s *t-*test.

GABA is the main product of the GAD system and therefore measurement of its production could give insights into how the systems compare in these strains. To this end stationary phase cultures of both strains were challenged to mild acidic pH for one hour and both the extracellular and intracellular GABA (GABA_e_/GABA_i_) production was measured. Previous work has indicated that EGD-e and therefore, EGDm does not produce GABA_e_ while 10403S produces both GABA_e_ and GABA_i_ [14]. As expected, GABA_e_ was not produced here by any EGDm strain in response to low pH (Fig. 3**a**). After 1 h at pH 4.0 however EGDm produced over 5 mM GABA_i_. A finding worth documenting was that EGDm produced higher concentrations of GABA_i_ in response to acidification than our previously studied EGD-e strain (data not shown). Since the parent EGD-e strains came from separate laboratories it may be that some mutation had arisen in one of the lines that impacts on the GAD system. Deletion of either *gadD1* or *gadD2* however did not affect GABA_i_ production compared to the parent strain (Fig. 3**b**). GABA production in EGDm Δ*gadD3* however was almost abolished, with production just above detectable levels for the assay (0.45 mM; Fig. 3**a**). In exponential phase no differences in GABA levels were detected between EGDm and the corresponding *gadD* mutants; indeed GABA levels were at or below the detection limit of the assay in these cultures (Fig. S2). Unlike EGDm, stationary phase cultures of 10403S produced both GABA_i_ (2.72 mM) and GABA_e_ (3.35 mM) after acid treatment. Deletion of *gadD1* or *gadD3* did not significantly affect the acid-induced GABA production. 10403S Δ*gadD2* did have a significant decrease in both GABA_i_ (0.64 mM; Fig. 3**b**) and GABA_e_ (0.75 mM; Fig. 3**a**). A similar effect on GABA production was observed for the Δ*gadD2* mutant (Fig. S2). Thus the *gadD* gene with the highest transcript levels in each strain also contributed the most to GABA production in that strain; *gadD3* for EGDm and *gadD2* for 10403S.

### Expression of the GAD system is pH-independent

**Figure 4:**
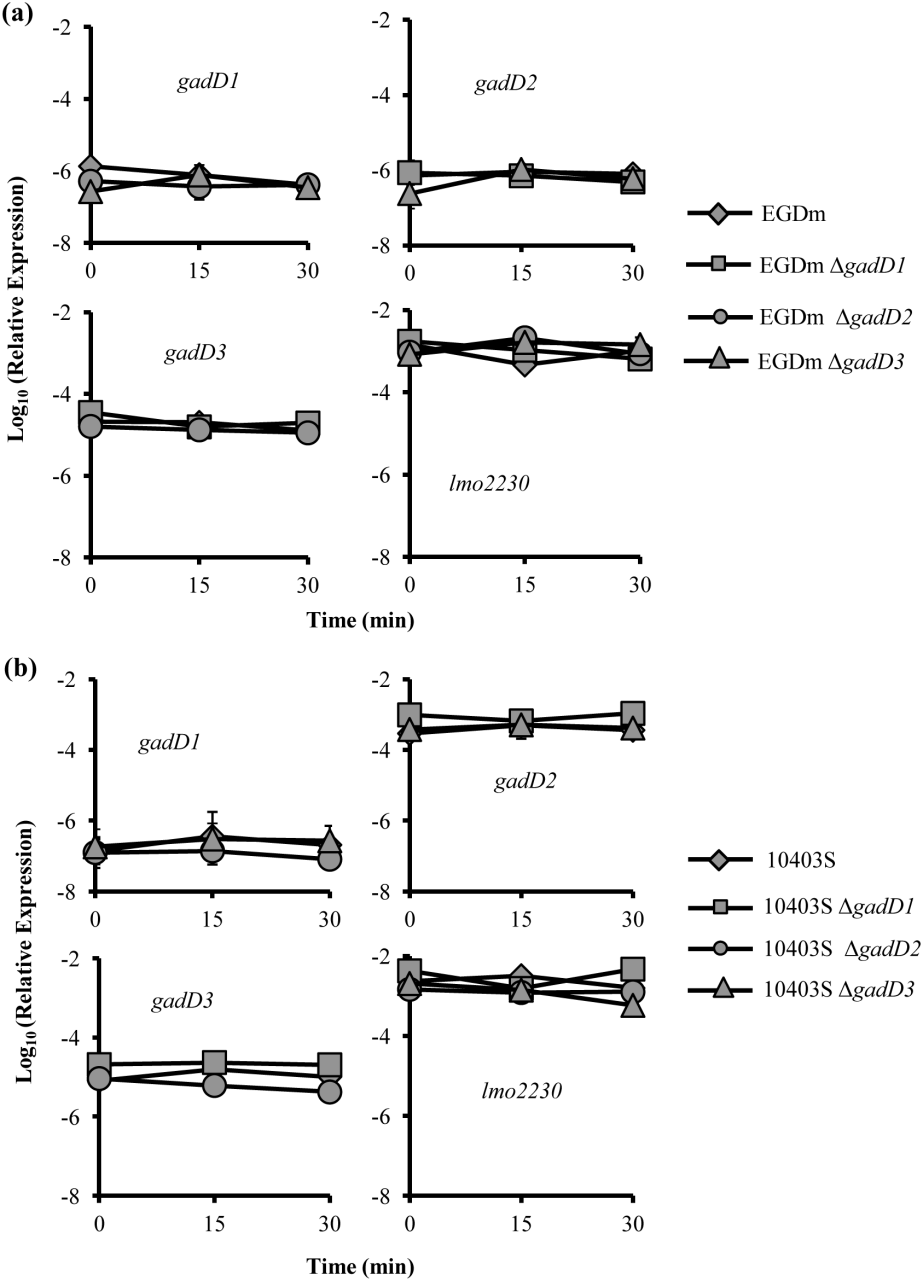
Relative expression of *gad* genes in response to acid treatment. Expression of *gadD1*, *gadD2*, *gadD3* and *lmo2230* relative to expression of the 16S rRNA gene prior to, 15 min and 30 min after exposure in BHI broth to pH 4.0 (EGDm **(a)**) or pH 3.5 (10403S **(b)**). Error bars represent the standard error in the mean of 3 independent biological repeats. Differences found to be significant between the genes at any time-point for each strain are shown with * Significance was determined where p <0.05 as determined by a Student’s *t*-test.

To determine the transcriptional response of EGDm and 10403S to both low pH exposure and GAD system mutation, the expression levels of all three decarboxylase genes was compared to the expression of 16S rRNA in each strain. Overall expression of the *gadD* genes did not change in EGDm in response to low pH (Fig. 4**a**). The expression of *gadD3* remained higher than both *gadD1* and *gadD2* in the parent EGDm strain and EGDm Δ*gadD1* and EGDmΔ*gadD2* throughout the course of the acid challenge. In 10403S, the relative expression of *gadD2* was significantly greater than the other genes at stationary phase (Fig 2. **d**). Deletion of any of the *gadD* genes did not affect the expression of the remaining two in stationary phase. The expression of the *gadD* genes did not appear to change in response to acid challenge for any of the four 10403S strains (Fig. 4**b**). σ^B^ activity, which was measured indirectly by recording the levels of the known σ^B^-dependent gene *lmo2230* (which encodes a putative arsenate reductase) [2], appeared to remain stable in all strains throughout the acid challenge (Fig. 4**a** bottom right). Overall it appeared that regardless of pH *gadD3* was the dominant GAD transcript in EGDm while for 10403S it was *gadD2.*

### Deletion of *gadD3* together with either *gadD1* or *gadD2* reduces acid tolerance seen in single mutants and abolishes GABA production

**Figure 5:**
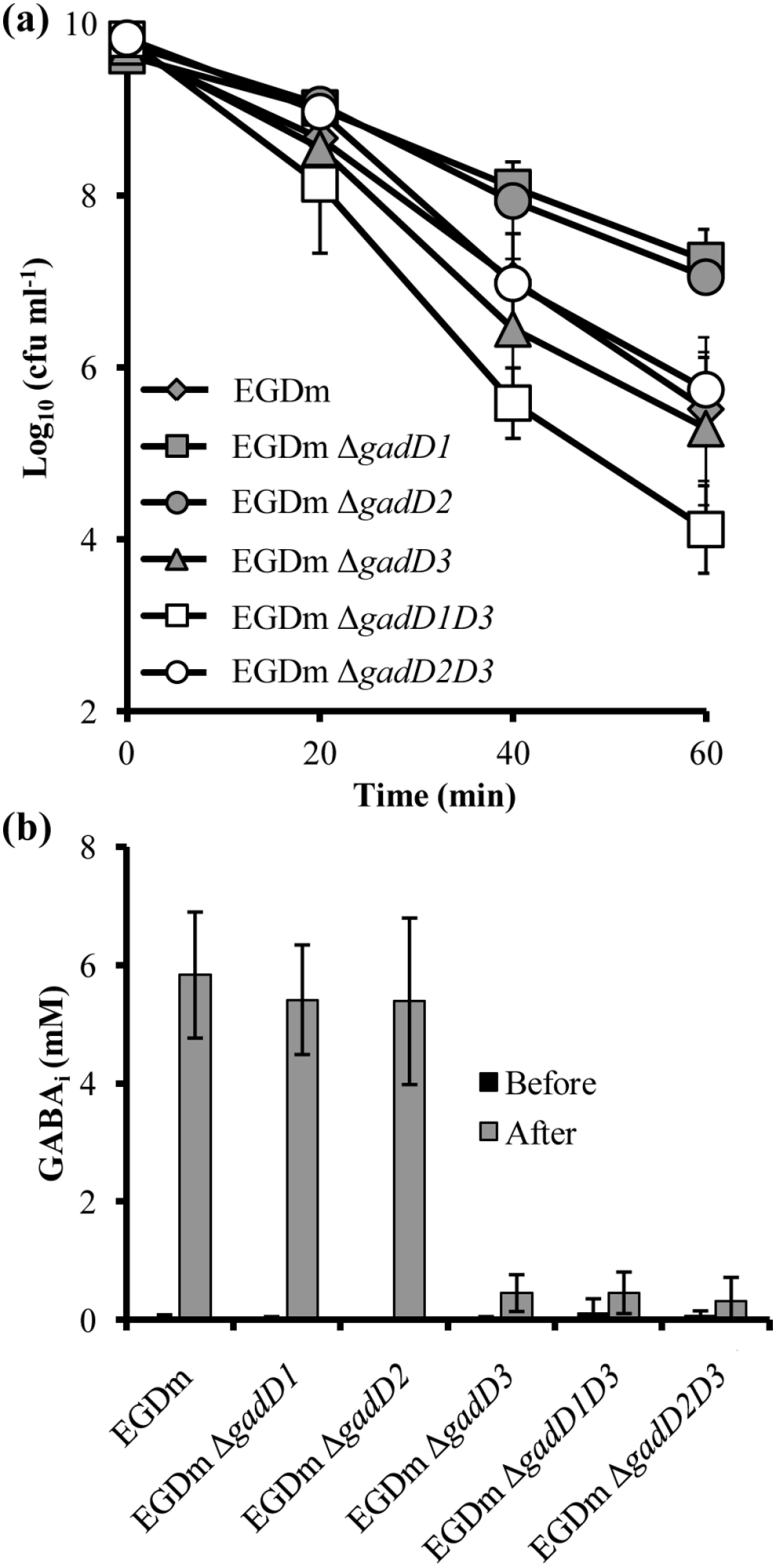
Acid survival and GABA_i_ production of *L. monocytogenes* EGDm double GAD system mutants indicates a key role for *gadD3*. (**a**) Stationary phase EGDm *gadD* mutants were acidified to pH 2.5 with 3 M HCl in BHI broth. Cell counts were taken every 20 min. Values are the means of data from three individual cultures, with the cell counts for each culture being the means of counts from three platings. Error bars represent the standard deviation from the mean value for each time-point. (**b**) Stationary phase EGDm gad mutants were acidified (grey) or not acidified (black) with 3 M HCl to pH 4.0 and GABA_i_ accumulation was quantified. Error bars represent the standard deviation from the mean of three independent biological replicates.

To account for the increase in acid survival seen in both EGDm Δ*gadD1* and EGDm Δ*gadD2* (Fig. 2**a**) double knockout mutant were constructed and tested for survival to exposure in pH 2.5 in BHI. EGDm Δ*gadD1D3* counts reduced at a faster rate than EGDm and was about 1-log cycle lower than the wt after 60 min (Fig. 5**a**). The presence of *gadD2* on its own did not appear to protect the strain as well as *gadD1* alone. Deletion of both *gadD2* and *gadD3* however followed a pattern of survival similar to the wild-type but reduced compared to the single knockout of *gadD1* (Fig. 5**a**). Overall it appeared that removing the *gadD3* gene from EGDm Δ*gadD1* or EGDm Δ*gadD2* negatively impacted on the increased acid survival seen with these strains in stationary phase. In exponential phase the Δ*gadD1D3* mutant was also found to have increased sensitivity to acid compared to either single deletion alone (Fig. S1**a**), further highlighting the importance of *gadD3* to this strain.

GABA_i_ was measured in stationary phase for each strain after exposure to pH 4.0. The mutants carrying double deletions both failed to produce GABA_i_ in response to exposure to the low pH (Fig. 5**b**). Possession of either *gadD1* or *gadD2* alone did not bestow an ability to produce GABA. As EGDm is unable to utilise the GADe system no GABA_e_ was produced by any of these strains in response to acid treatment (data not shown).

### EGDm *gadD* mutants survive gastric passage in mice

**Figure 6:**
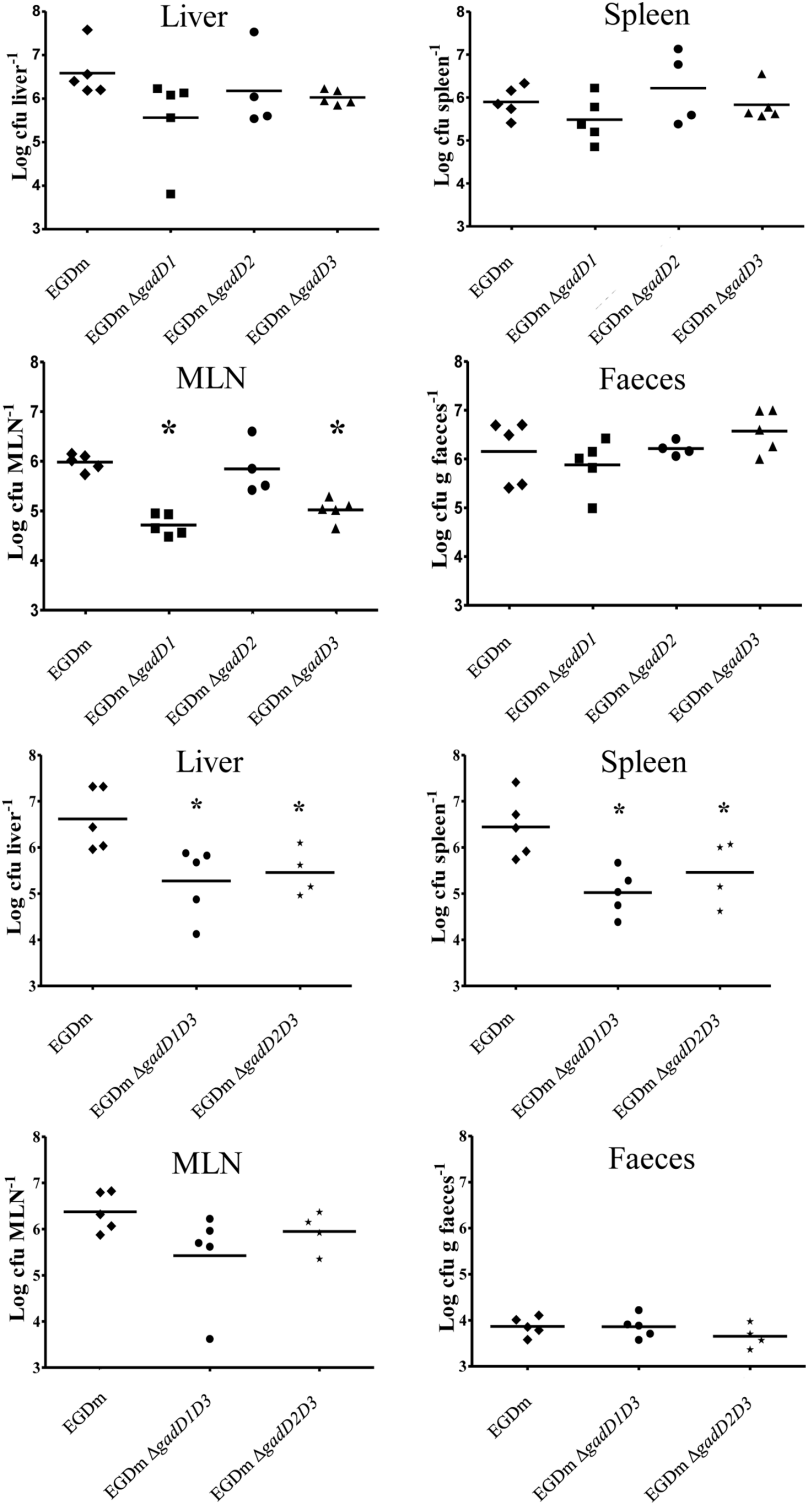
Infection of Balb/C mice with EGDm GAD system mutants. Plate counts of surviving EGDm GAD system mutants 3 days post infection from female Balb/C mice (n = 5). Isolated from the liver, spleen, mesenteric lymph node (MLN) and faeces. Significant differences (*) between wild-type and mutants were determined using one-way ANOVA.

In order to analyse the role that the GAD_i_ system plays *in vivo*, survival through a live animal gastric passage was carried out. Three days post oral inoculation of female Balb/C mice with each of the EGDm *gad* mutants, the animals were sacrificed and dissemination of the strains was analysed. Counts ranged from 6.50×10^3^ to 3.80×10^7^ cfu ml^−1^ and were similar in the liver, spleen and intestinal content for all single deletion strains and the respective wild-type. The counts for EGDm Δ*gadD1* and EGDm Δ*gadD3* however were significantly lower in the mesenteric lymph node (MLN) compared to the wild-type (1-log; Fig. 6). Double deletion of either *gadD1* with *gadD3* or *gadD2* with *gadD3* resulted in reduced counts from both the liver and spleens of mice 3 days post infection. These data suggest that the GAD_i_ system plays a role within the host during the development of an infection and is essential for full virulence potential following oral infection of mice.

### Growth of *L. monocytogenes* GAD system mutants in human THP-1 macrophages

**Figure 7:**
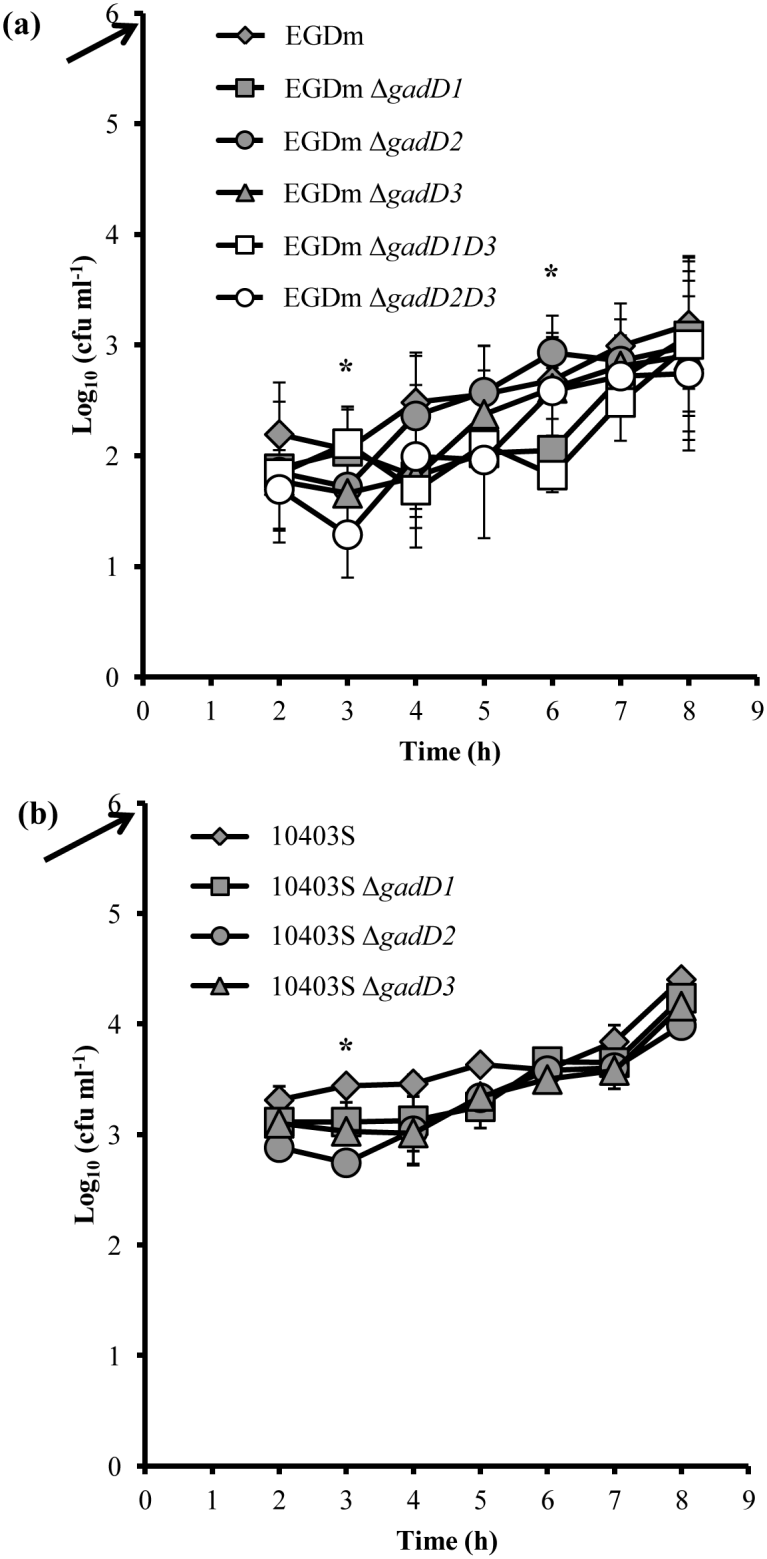
Growth of GAD system mutants in THP-1 macrophages. Growth of EGDm (**a**) and 10403S (**b**) GAD system mutants inside THP-1 macrophages over 7 h. Counts are recorded 2 h post co-incubation of THP-1 with bacteria at an MOI of 10 (10^6^ bacteria; black arrow). Error bars represent the standard deviation from the mean of at least 4 biological replicates for each strain and time-point. Significant differences (*; *p* <0.05) were determined using one-way ANOVA.

In order to cause infection *L. monocytogenes* must be able to survive inside phagocytic macrophages. The environment that the bacteria encounter inside these cells is reported to be acidic [25] and thus we investigated the role that the GAD system may play in survival. None of the EGDm mutants showed a significant difference in uptake rate by THP-1 cells compared to the wild type after 2 h (Fig. 7**a**). Furthermore, there was no significant difference seen in the ability of any strain to grow inside the THP-1 cells over a 7 h time-course. EGDm Δ *gadD1* and EGDm Δ*gadD2* did however show a significant lag until 5 h but eventually reached similar numbers to both EGDm and the *gadD3* mutant (Fig. 7**a**). Similarly, 10403S and its isogenic GAD system mutants grew inside THP-1 macrophages with only an apparent lag seen for 10403S Δ*gadD2* after 3 h. Otherwise no significant differences were seen between the wild-type and mutants for the duration of the experiment.

## Discussion

Previous studies with *L. monocytogenes* have shown that strains can vary greatly in terms of their ability to cope with low pH [12], [14]. Here the GAD system, one of the major mechanisms for acid tolerance was compared between two commonly used reference strains EGDm (murinised EGD-e) and 10403S. The survival of 10403S during exposure to low pH is significantly greater than EGDm (Fig. 2) and this correlates with an ability of 10403S to utilise both GAD_i_ and GAD_e_ in response to low pH. Despite possessing the genes for a putative glutamate/GABA antiporter, EGDm does not export GABA. This may be due to the fact that the amino acid sequences of GadT2 are different at four positions between 10403S and EGDm (V409I, V419I, M438T, I441M, respectively) unlike GadT1 which varies by only a single amino acid (T196K). However, efforts to clone the *gadT2* gene from 10403S into EGDm were unsuccessful in inducing GABA_e_ production in EGDm (data not shown). Further studies will be required to test whether expression of *gadT2* together with *gadD2* from 10403S (i.e. the entire *gadT2D2* operon) can induce GABA_e_ production in an EGD-e background.

As EGDm appears to be solely reliant upon intracellular decarboxylation (GAD_i_), the three genes encoding glutamate decarboxylases (*gadD1, gadD2* and *gadD3*) were deleted. Somewhat surprisingly, deletion of *gadD1* or *gadD2* improved the ability of the strain to survive low pH during stationary phase (Fig. 2**a**), as there was no apparent increase in GABA production, the increased acid resistance may be due to the action of other acid tolerance mechanisms that were not investigated as part of this study. The increase in acid tolerance was comparable to the levels of resistance seen for 10403S, which is one of the most resistant strains of *L. monocytogenes* [26] (Fig. 2**b**). None of the single deletions were seen to reduce the bacterium’s ability to survive at low pH, showing that each is dispensable for wild-type acid tolerance levels in this strain background. However, deletion of both *gadD1* and *gadD3* together did prevent the increase in survival seen for EGDm Δ*gadD1* and this double mutant strain, EGDm Δ*gadD1D3,* was more acid sensitive than EGDm (Fig. 5**a** & Fig. S1**a**). The deletion of *gadD3* along with *gadD2* also appeared to prevent the increase in stationary phase acid tolerance seen in the Δ*gadD2* mutant however this strain was not as sensitive as EGDm Δ*gadD1D3* suggesting that *gadD1* has a greater role to play in acid tolerance than *gadD2* in this strain. Measurements of GABA_i_ across the strains in response to acid, indicated that only EGDm Δ*gadD3* was impaired in an ability to produce GABA_i_ during stationary phase. Taken together with the acid survival data, it would appear that this decarboxylase plays a more important role in the GAD system of this strain compared with the remaining two. Interestingly, a different effect was observed in 104033S after the deletion of the *gadD* genes. Here deletion of *gadD2* negatively impacted on survival during both stationary phase and mid-exponential phase growth (Fig. 2**b** & Fig. S1**b**). In 10403S the *gadD2* deletion was also accompanied by a reduction in both GABA_i_ and GABA_e_ (Fig. 3 & Fig. S2). It would appear that failure to produce GABA in this strain impacted negatively on its ability to survive at low pH. As *gadD2 gadT2* are part of the same operon it is not surprising that both GABA_i_ and GABA­_e_ production was affected. The remaining GadT1D1 system apparently could not compensate for the loss of GadT2D2 activity in this strain.

An examination of the transcriptional response for each of the strains in response to acid confirms the differential importance of either *gadD2* or *gadD3* for 10403S and EGDm, respectively. The *gadD2* transcript was the most abundant of the three in 10403S in stationary phase cultures, whereas *gadD3* was dominant in the EGDm background (Fig. 4). Overall, neither strain displayed an alteration in *gadD* gene expression in response to the pH treatment. This may be due to the fact that the cultures have already reached stationary phase and therefore were expressing each gene to a maximal level. Although little is still known about the transcriptional regulation of the GAD system in *L. monocytogenes*, it is known that both the *gadT2D2* operon and *gadD3* are at least partially under the control of σ^B^. Using *lmo2230* (encoding a putative arsenate reductase) as a reporter of σ^B^ activity [2], [27], there was clearly no change in σ^B^ activity over the course of the acid treatment or as a result of the deletions in the *gadD* genes. As shown previously [2], [27] σ^B^ is fully active in stationary phase and additional stress doesn’t enhance its activity beyond that level. This might indicate that the cells already possess functional GAD system proteins prior to acid shock and that regulation of the GAD system occurs post transcriptionally.

While much of the previous work undertaken on the GAD system in *L. monocytogenes* has focused on *in vitro* models and synthetic gastric fluid, the role of the system *in vivo* has not been addressed previously. In fact there is very little evidence in any bacterial pathogen showing that the GAD system is important for survival in the host. Work with *Brucella microti* has however shown that deletion of the GAD system reduced counts in mice after oral inoculation [28]. In our study we also found a significant role for the GAD system in the virulence of EGDm in a mouse infection model.

Due to the extensive use of the EGD-e strain as a model intracellular pathogen [29]–[31], we focused upon the role of the GAD_i_ system in an oral infection model that employs murinised EGDm strain to enhance the progression of invasive disease via the GI tract [20]. A previous study identifying genes expressed by *L. monocytogenes* in response to *Lactobacilli* within the mouse gut showed that all three gene systems (*gadD1T1, gadD2T2* and *gadD3*) had significantly increased expression in this environment [32]. This suggests that the GAD system is actively induced within the host GI tract. In our functional study single *gadD* deletion mutants were not affected in their ability to infect the liver and spleen of mice following oral infection. However the two double mutants displayed significantly reduced levels of infection, indicating that a complete GAD system is required for full infection. This correlates with *in vitro* survival assays with these mutants, particularly EGDm Δ*gadD1D3,* which displays a greater sensitivity to low pH than the single mutants and exhibits a significant virulence defect in our model system. It is important to note that the pH in the stomach of Balb/C mice can be as high as pH 4.04 [33] and the gastric acidity of this particular animal host may have implications for the survival of these *gad* mutant strains. However, the GAD system is likely to play a more important role in hosts that possess stomachs with higher acidity. It will also be important to investigate the role of the GAD_e_ system in virulence by using a *L. monocytogenes* strain capable of producing GABA_e_.

Subsequently, we investigated another stage in the virulence process of *L. monocytogenes*; the intracellular cycle. In both EGDm and 10403S, the GAD system does not appear to play a major role in survival within human derived macrophages. This was largely expected since the pH inside bone marrow derived macrophages after compartmentalisation of *L. monocytogenes* is about pH 5.5 [25] while induction of the GAD system in *L. monocytogenes* normally occurs below pH 4.5 [17]. Interestingly, although GadD1T1 is known to play a role in growth at mild acidic conditions [16] similar to those occurring within macrophages no major effect of these proteins was documented within macrophages.

Overall, the GAD system in *L. monocytogenes* appears to show a clear divergence in functionality between these two well-studied strains. The strains appear to have adopted the use of either a GAD_i_ system or a combined GAD_i_/GADe system, which likely reflects their unique evolutionary histories. The strain which possessed both GAD_i_ and GAD_e_, 10403S, displayed a more acid resistant phenotype suggesting that a functional antiport is highly beneficial. In contrast, the strain which utilised only GAD_i_ did not appear to have a sole reliance on any of its three isoforms of decarboxylases for acid tolerance, indicating a more robust system. From comparing these two strains it is clear that functional divergence of physiologically important pathways can readily occur and further highlights the importance of inter-strain comparisons in addressing the biological significance of any pathway.

## Supporting Information

Figure S1 **Acid survival of** ***L. monocytogenes gad*** **mutants.** Mid-log phase EGDm **(a)** and 10403S **(b)** Δ*gad* mutants were challenged at pH 3.0. Cell counts were taken every 20 min. Values are the means of data from three individual cultures, with the cell counts for each culture being the means of counts from three platings. Error bars represent the standard error from the mean value of three individual biological repeats.
(TIF)

Figure S2 **GABA production from** ***L. monocytogenes gad*** **mutants.** **(a)** Production of GABA_e_ by EGDm and 10403S gad mutants with (grey) or without (black) 1 h exposure to acid at pH 4.0 (EGDm) or pH 3.5 (10403S). **(B)** Production of GABA_i_ by EGDm and 10403S *gadD* mutants with (grey) or without (black) 1 h exposure to acid at pH 4.0 (EGDm) or pH 3.5 (10403S). Dashed horizontal lines indicate the detection limits for GABA in each experiment. Error bars represent the standard deviation from the mean of three individual biological repeats for each sample.
(TIF)

## References

[1] Abram F, Starr E, Karatzas KAG, Matlawska-Wasowska K, Boyd A, et al (2008) Identification of components of the sigma B regulon in *Listeria monocytogenes* that contribute to acid and salt tolerance. Appl Environ Microbiol 74: 6848–6858. 18806006 10.1128/AEM.00442-08 PMC2583506

[2] Utratna M, Shaw I, Starr E, O’Byrne CP (2011) Rapid, transient, and proportional activation of σ^B^ in response to osmotic stress in *Listeria monocytogenes.* . Appl Environ Microbiol 77: 7841–7845. 21890665 10.1128/AEM.05732-11 PMC3209154

[3] Cole MB, Jones M V, Holyoak C (1990) The effect of pH, salt concentration and temperature on the survival and growth of *Listeria monocytogenes* . J Appl Microbiol 69: 63–72. 10.1111/j.1365-2672.1990.tb02912.x 2118897

[4] Karatzas KAG, Wouters JA, Gahan CGM, Hill C, Abee T, et al (2003) The CtsR regulator of *Listeria monocytogenes* contains a variant glycine repeat region that affects piezotolerance, stress resistance, motility and virulence. Mol Microbiol 49: 1227–1238. 12940983 10.1046/j.1365-2958.2003.03636.x

[5] Wemekamp-Kamphuis HH, Wouters JA, Leeuw PPLA De, Hain T, Chakraborty T, et al (2004) Identification of Sigma Factor B -Controlled Genes and Their Impact on Acid Stress, High Hydrostatic Pressure, and Freeze Survival in *Listeria monocytogenes* EGD-e. Appl Environ Microbiol 70: 3457–3466. 15184144 10.1128/AEM.70.6.3457-3466.2004 PMC427741

[6] Walker SJ, Archer P, Banks JG (1990) Growth of *Listeria monocytogenes* at refrigeration temperatures. J Appl Bacteriol 68: 157–162. 2108109 10.1111/j.1365-2672.1990.tb02561.x

[7] Young KM FP (1993) Acetic, lactic and citric acids and pH inhibition of *Listeria monocytogenes* Scott A and the effect on intracellular pH. J Appl Bacteriol 74: 515–520. 8486558

[8] Heavin SB, Brennan OM, Morrissey JP, O’Byrne CP (2009) Inhibition of *Listeria monocytogenes* by acetate, benzoate and sorbate: weak acid tolerance is not influenced by the glutamate decarboxylase system. Lett Appl Microbiol 49: 179–185. 19422474 10.1111/j.1472-765X.2009.02634.x

[9] Ryan S, Begley M, Gahan CGM, Hill C (2009) Molecular characterization of the arginine deiminase system in *Listeria monocytogenes*: Regulation and role in acid tolerance. Environ Microbiol 11: 432–445. 19196274 10.1111/j.1462-2920.2008.01782.x

[10] Cotter PD, Gahan CGM, Hill C (2000) Analysis of the role of the *Listeria monocytogenes* F_0_F_1_ -ATPase operon in the acid tolerance response. Int J Food Microbiol 60: 137–146. 11016603 10.1016/s0168-1605(00)00305-6

[11] Davis MJ, Coote PJ, O’Byrne CP (1996) Acid tolerance in *Listeria monocytogenes*: the adaptive acid tolerance response (ATR) and growth-phase-dependent acid resistance. Microbiology 142: 2975–2982. 8885415 10.1099/13500872-142-10-2975

[12] Cotter PD, Gahan CGM, Hill C (2001) A glutamate decarboxylase system protects *Listeria monocytogenes* in gastric fluid. Mol Microbiol 40: 465–475. 11309128 10.1046/j.1365-2958.2001.02398.x

[13] Tsai M, Mccarthy P, Miller C (2013) Substrate selectivity in glutamate-dependent acid resistance in enteric bacteria. Proc Natl Acad Sci 110: 5898–5902. 23530225 10.1073/pnas.1301444110 PMC3625338

[14] Karatzas KAG, Suur L, O’Byrne CP (2012) Characterization of the Intracellular Glutamate Decarboxylase System: Analysis of Its Function, Transcription, and Role in the Acid Resistance of Various Strains of *Listeria monocytogenes.* . Appl Environ Microbiol 78: 3571–3579. 22407692 10.1128/AEM.00227-12 PMC3346379

[15] Glaser P, Frangeul L, Buchrieser C, Rusniok C, Amend A, et al (2001) Comparative genomics of Listeria species. Science (80- ) 294: 849–852. 10.1126/science.1063447 11679669

[16] Cotter PD, Ryan S, Gahan CGM, Hill C (2005) Presence of GadD1 glutamate decarboxylase in selected *Listeria monocytogenes* strains is associated with an ability to grow at low pH. Appl Environ Microbiol 71: 2832–2839. 15932974 10.1128/AEM.71.6.2832-2839.2005 PMC1151821

[17] Karatzas KAG, Brennan O, Heavin S, Morrissey J, O’Byrne CP (2010) Intracellular Accumulation of High Levels of γ-Aminobutyrate by *Listeria monocytogenes* 10403S in Response to Low pH: Uncoupling of γ-Aminobutyrate Synthesis from Efflux in a Chemically Defined Medium. Appl Environ Microbiol 76: 3529–3537. 20400565 10.1128/AEM.03063-09 PMC2876448

[18] Horton RM, Ho SN, Pullen JK, Hunt HD, Cai Z, et al (1993) Gene splicing by overlap extension: tailor-made genes using the polymerase chain reaction. Methods Enzymol 217: 270–279. 8474334 10.1016/0076-6879(93)17067-f

[19] O’Byrne CP, Feehily C, Ham R, Karatzas KAG (2011) A modified rapid enzymatic microtiter plate assay for the quantification of intracellular γ-aminobutyric acid and succinate semialdehyde in bacterial cells. J Microbiol Methods 84: 137–139. 21044648 10.1016/j.mimet.2010.10.017

[20] Monk IR, Casey PG, Hill C, Gahan CGM (2010) Directed evolution and targeted mutagenesis to murinize *Listeria monocytogenes* internalin A for enhanced infectivity in the murine oral infection model. BMC Microbiol 10: 318. 21144051 10.1186/1471-2180-10-318 PMC3016325

[21] Tsuchiya S, Yamabe M, Yamaguchi Y, Kobayashi Y, Konno T, et al (1980) Establishment and characterization of a human acute monocytic leukemia cell line (THP-1). Int J Cancer 26: 171–176. 6970727 10.1002/ijc.2910260208

[22] Monk IR, Gahan CGM, Hill C (2008) Tools for functional postgenomic analysis of *Listeria monocytogenes* . Appl Environ Microbiol 74: 3921–3934. 18441118 10.1128/AEM.00314-08 PMC2446514

[23] Hersh BM, Farooq FT, Barstad DN, Blankenhorn DL, Slonczewski JL (1996) A glutamate-dependent acid resistance gene in *Escherichia coli* . J Bacteriol 178: 3978–3981. 8682809 10.1128/jb.178.13.3978-3981.1996 PMC232665

[24] Bécavin C, Bouchier C, Lechat P, Archambaud C, Creno S, et al. (2014) Comparison of Widely Used *Listeria monocytogenes* Strains EGD, 10403S, and EGD-e Highlights Genomic Differences Underlying Variations in Pathogenicity. MBio 5. 10.1128/mBio.00969-14 PMC3977354 24667708

[25] De Chastellier C, Berche P (1994) Fate of *Listeria monocytogenes* in murine macrophages: evidence for simultaneous killing and survival of intracellular bacteria. Infect Immun 62. 10.1128/iai.62.2.543-553.1994 PMC186140 8300212

[26] Feehily C, O’Byrne CP, Karatzas KAG (2013) Functional γ-Aminobutyrate Shunt in *Listeria monocytogenes*: role in acid tolerance and succinate biosynthesis. Appl Environ Microbiol 79: 74–80. 23064337 10.1128/AEM.02184-12 PMC3536111

[27] Utratna M, Cosgrave E, Baustian C, Ceredig R, O’Byrne CP (2014) Effects of Growth Phase and Temperature on σ^B^ Activity within a *Listeria monocytogenes* Population: Evidence for RsbV-Independent Activation of σ^B^ at Refrigeration Temperatures. Biomed Res Int. 10.1155/2014/641647 PMC3964741 24734238

[28] Occhialini A, Pilar M, Bagüés J De, Saadeh B, Bastianelli D, et al (2012) The Glutamic Acid Decarboxylase System of the New Species *Brucella microti* Contributes to Its Acid Resistance and to Oral Infection of Mice. J Infect Dis 206: 1424–1432. 22930809 10.1093/infdis/jis522

[29] Toledo-Arana A, Dussurget O, Nikitas G, Sesto N, Guet-Revillet H, et al (2009) The Listeria transcriptional landscape from saprophytism to virulence. Nature 459: 950–956. 19448609 10.1038/nature08080

[30] Joseph B, Przybilla K, Stuhler C, Schauer K, Slaghuis J, et al (2006) Identification of *Listeria monocytogenes* genes contributing to intracellular replication by expression profiling and mutant screening. J Bacteriol 188: 556. 16385046 10.1128/JB.188.2.556-568.2006 PMC1347271

[31] Chatterjee SS, Hossain H, Otten S, Kuenne C, Kuchmina K, et al (2006) Intracellular Gene Expression Profile of *Listeria monocytogenes* . Infect Immun 74: 1323. 16428782 10.1128/IAI.74.2.1323-1338.2006 PMC1360297

[32] Archambaud C, Nahori M, Soubigou G, Bécavin C, Laval L, et al (2012) Impact of lactobacilli on orally acquired listeriosis. Proc Natl Acad Sci 109: 16684–16689. 23012479 10.1073/pnas.1212809109 PMC3478606

[33] McConnell EL, Basit AW, Murdan S (2008) Measurements of rat and mouse gastrointestinal pH, fluid and lymphoid tissue, and implications for *in vivo* experiments. J Pharm Pharmacol 60: 63–70. 18088506 10.1211/jpp.60.1.0008

[34] Smith K, Youngman P (1992) Use of a new integrational vector to investigate compartment-specific expression of the *Bacillus subtilis spollM* gene. Biochimie 74: 705–711. 1391050 10.1016/0300-9084(92)90143-3

