# Supplementary figures and images for: Divergent Evolution of the Activity and Regulation of the Glutamate Decarboxylase Systems in Listeria monocytogenes EGD-e and 10403S: Roles in Virulence and Acid Tolerance
Source: PLoS One. 2014 Nov 11;9(11):e112649. doi: 10.1371/journal.pone.0112649 (PMC4227838; doi:10.1371/journal.pone.0112649)

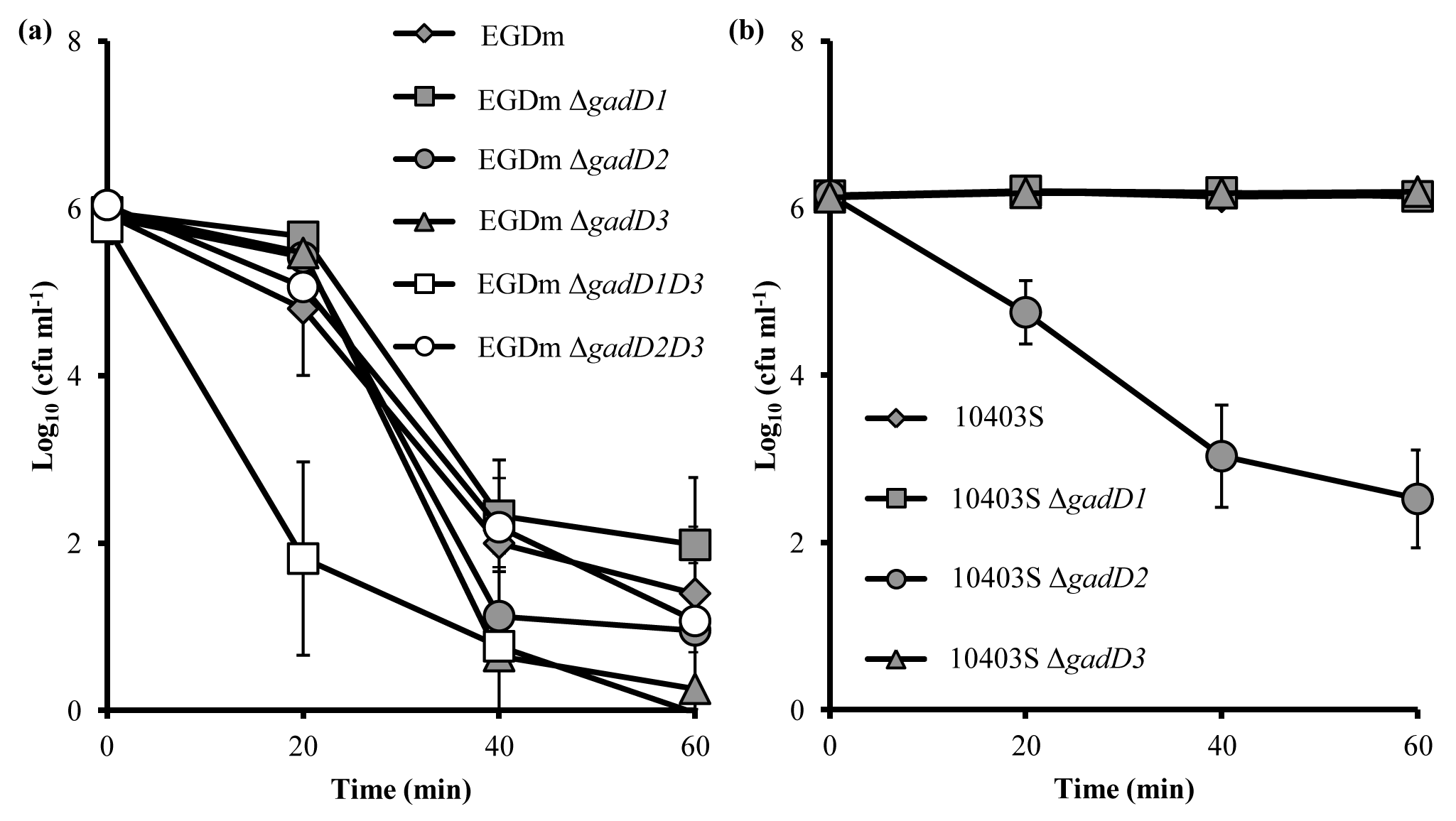

Supplement: Figure S1 — Acid survival of L. monocytogenes gad mutants. Mid-log phase EGDm (a) and 10403S (b) Δgad mutants were challenged at pH 3.0. Cell counts were taken every 20 min. Values are the means of data from three individual cultures, with the cell counts for each culture being the means of counts from three platings. Error bars represent the standard error from the mean value of three individual biological repeats. (TIF) [file pone.0112649.s001.tif]

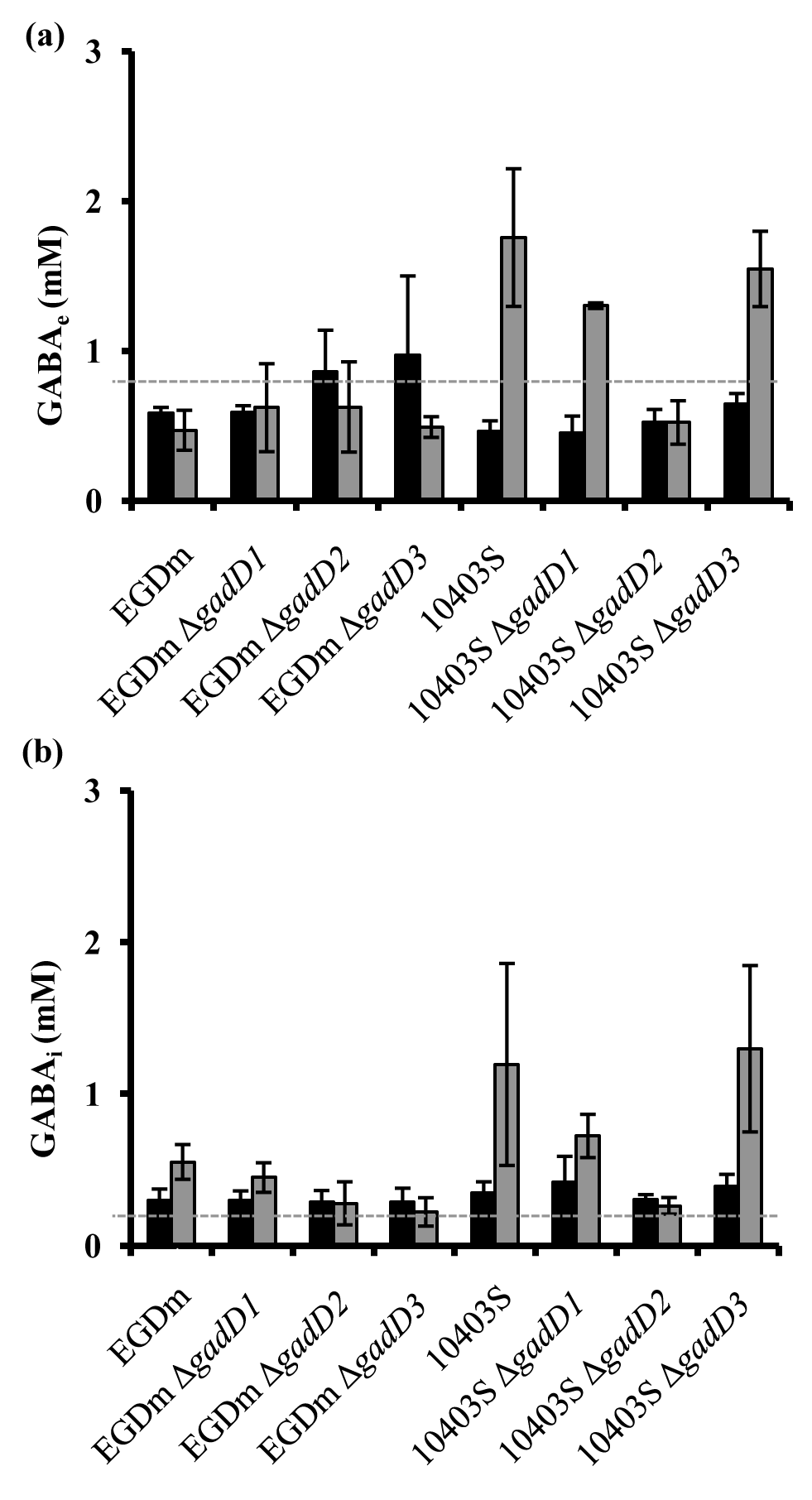

Supplement: Figure S2 — GABA production from L. monocytogenes gad mutants. (a) Production of GABAe by EGDm and 10403S gad mutants with (grey) or without (black) 1 h exposure to acid at pH 4.0 (EGDm) or pH 3.5 (10403S). (B) Production of GABAi by EGDm and 10403S gadD mutants with (grey) or without (black) 1 h exposure to acid at pH 4.0 (EGDm) or pH 3.5 (10403S). Dashed horizontal lines indicate the detection limits for GABA in each experiment. Error bars represent the standard deviation from the mean of three individual biological repeats for each sample. (TIF) [file pone.0112649.s002.tif]
